# Adhesion of *Escherichia Coli* to Nanostructured Surfaces and the Role of Type 1 Fimbriae

**DOI:** 10.3390/nano10112247

**Published:** 2020-11-12

**Authors:** Pawel Kallas, Håvard J Haugen, Nikolaj Gadegaard, John Stormonth-Darling, Mats Hulander, Martin Andersson, Håkon Valen

**Affiliations:** 1 Department of Biomaterials, Institute of Clinical Dentistry, University of Oslo, 0455 Oslo, Norway; pawel.kallas@odont.uio.no; 2 School of Engineering, University of Glasgow, G12 8QQ Glasgow, UK; Nikolaj.Gadegaard@glasgow.ac.uk (N.G.); John.Stormonth-Darling@glasgow.ac.uk (J.S.D.); 3 Department of Chemistry and Chemical Engineering, Chalmers University of Technology, 412 58 Göteborg, Sweden; mats.hulander@chalmers.se (M.H.); martin.andersson@chalmers.se (M.A.); 4 Nordic Institute of Dental Materials, 0855 Oslo, Norway; hakon.valen@niom.no

**Keywords:** nanostructured surface, injection molding, anti-adhesive, *E. coli*, fimbriae, anti-bacterial, biomaterial-associated infections (BAI)

## Abstract

Bacterial fimbriae are an important virulence factor mediating adhesion to both biotic and abiotic surfaces and facilitating biofilm formation. The expression of type 1 fimbriae of *Escherichia coli* is a key virulence factor for urinary tract infections and catheter-associated urinary tract infections, which represent the most common nosocomial infections. New strategies to reduce adhesion of bacteria to surfaces is therefore warranted. The aim of the present study was to investigate how surfaces with different nanotopography-influenced fimbriae-mediated adhesion. Surfaces with three different nanopattern surface coverages made in polycarbonate were fabricated by injection molding from electron beam lithography nanopatterned templates. The surfaces were constructed with features of approximately 40 nm width and 25 nm height with 100 nm, 250 nm, and 500 nm interspace distance, respectively. The role of fimbriae type 1-mediated adhesion was investigated using the *E. coli wild type BW25113* and Δ*fimA* (with a knockout of major pilus protein FimA) and Δ*fimH* (with a knockout of minor protein FimH) mutants. For the surfaces with nanotopography, all strains adhered least to areas with the largest interpillar distance (500 nm). For the *E. coli wild type*, no difference in adhesion between surfaces without pillars and the largest interpillar distance was observed. For the deletion mutants, increased adhesion was observed for surfaces without pillars compared to surfaces with the largest interpillar distance. The presence of a fully functional type 1 fimbria decreased the bacterial adhesion to the nanopatterned surfaces in comparison to the mutants.

## 1. Introduction

Bacterial adhesion, colonization, and biofilm formation on medical devices are responsible for a large proportions of nosocomial infections, and biomaterial-associated infections are common post-operative [1,2,3,4]. Catheter-associated urinary tract infections (CAUTI) are the most common hospital acquired bacterial infection and are associated with increased mortality and morbidity [5]. The gram negative bacterium *Escherichia coli* (*E. coli*) is the most frequent cause of CAUTIs [6].

*E. coli* is naturally found in the intestinal flora of humans and animals [7] and includes both non-pathogenic and pathogenic strains [8,9,10]. Some *E. coli* strains express type 1 fimbria, a bacterial adhesion that has been reported to facilitate adhesion to other bacteria, host cells, and surfaces of medical devices [11]. Type 1 fimbria have a polymeric protein structure, made of FimA, FimF, FimG, and FimH monomers, with a tubular structure of 7 nm in width and 1–2 μm in length [12,13,14]. Expression of fimbriae has been reported to be an important virulence factor for uropathogenic *E. coli*, important for biofilm formation on abiotic surfaces [15]. *E. coli* expressing type 1 fimbriae was observed to contribute to catheter-associated urinary tract infections in a dynamic catheterized bladder model [16]. The biofilm may act as a natural barrier, protecting bacteria from antimicrobial treatment [17], and biofilms are associated with reduced susceptibility towards antimicrobial treatment [18].

Fimbrial adhesins can also be present in other Gram-negative species like *Serratia marcescens* or *Klebsiella pneumoniae*, where they a play crucial role in biofilm formation [19,20]. Fimbria is an important virulence factor for urinary tract infections [16], where *E. coli* has been reported to be the most prevalent pathogen [21,22,23,24,25], and the presence of fimbriae type 1 enhances the survival of *E. coli* in the urinary tract [26].

Finding new solutions in the fight against bacteria and, therefore, the need to develop medical devices that prevent adhesion of bacteria is highly desired. Controlling bacterial colonization can be done by modifying surface properties of medical devices, such as adhesion properties and coatings, surface roughness, and chemistry [27,28]. Changing surface topography, by altering the surface roughness and its complexity, can affect bacterial adhesion and therefore lower the risk of a potential infection [29,30,31,32,33]. Previous studies have shown that bacterial adhesion can also be influenced by the presence of metallic nanoparticles that show antimicrobial properties, such as such as silver (Ag), gold (Au), and zinc oxide (ZnO) [34]. Another type of nanoparticles that presents antibacterial and biocompatible features is silicon nanoparticles [35,36,37,38]. 

An important obstacle for nanomaterials is the step from scientific idea to a product on the market. Manufacturers need to have high scale, high quality production, and preservation of the desired properties of the nanomaterials [39]. Electron beam lithography (EBL) is a production technique that may enable high production with reproducible quality of materials with surface modifications in the nano-range [40].

The aim of our study was to investigate whether surfaces with different nanoscale topographies affected fimbriae-mediated adhesion of *E. coli*. The hypothesis was that presence of fimbriae has an effect on bacterial attachment to nanostructured surfaces. Electron beam lithography was used to produce controlled nanoscale surface topographies on a template for fabricating injection molded polycarbonate substrates as a model system with varying surface coverages. The higher the interspace distance between nanopillars, the lower the contact area between bacteria and nanopillars. If were to increase that distance, the individual bacteria would only have contact with a few pillars. Therefore, we decided to fabricate surfaces with interspace distances between nanopillars smaller than the actual size of the tested bacteria, namely 100, 250, and 500 nm. 

## 2. Materials and Methods 

### 2.1. Preparation of Nanostructured Surfaces

Nanostructured polymer surfaces were prepared by injection molding of patterns mastered by electron beam lithography on silicon masters. Silicon samples were solvent and oxygen plasma cleaned, dehydrated, and oxygen plasma treated again (this time for adhesion promotion) before immediately being spun on hydrogen silsesquioxane (HSQ) resist (FOx 12) at a thickness corresponding to the desired nanofeature height. Patterns were written in a Vistec VB6 UHR EWF EBL tool (Vistec Electron Beam GmbH, Jena, Germany) operating at 100 kV and developed in 25% tetramethylammonium hydroxide (TMAH) solution at 23 °C followed by rinsing in water and isopropyl alcohol (IPA). As it was not possible to spin HSQ films thinner than ~40 nm, the 20 nm features had to be defined by dry etching with the HSQ pattern, and the mask was subsequently removed by hydrofluoric acid (HF). Dry etching was performed in an STS ICP silicon etch tool using a mixed process and always preceded by a dilute HF dip (2000:1, 48% HF:RO water, by vol.) to prevent micromasking by native oxide. All chemicals were provided by Sigma-Aldrich, Saint-Louis, MO, USA.

Nanopatterns were then replicated using an UV-based nanoimprint lithography (UV-NIL) (EV Group, Sankt Florian am Inn, Austria) into the working stamp material. Silicon masters were first spin coated with the EVG anti-stick material, followed immediately by the working stamp material, which was UV cured against the master onto a carried foil by way of the NIL tool’s standard working stamp recipe. UV-NIL was performed using a custom-built pocket imprinter. Each working stamp replica was laser cut to the appropriate dimensions of the injection molding tool, as described in [41]. 

Injection molding was performed in an Engel Victory 28 hydraulic injection molding machine (Engel Austria GmbH, Schwertberg, Austria) to produce multiple polystyrene samples for biological experiments. Each surface consisted of 9 × 4 repetitions of pattern divided into three sections (total size 1 × 3 mm) with different surface coverages, namely 2.5, 3.5, and 20%. The total number of surface coverages produced was *n* = 972, although due to production process, some gradients were not included in the image analysis. Therefore, the total number of nanostructured surfaces with different coverages measured was 191.

### 2.2. Surface Characterization

The heights of the pillars were characterized with an atomic force microscope (Veeco Dimension 3100, Santa Barbara, CA, USA). AFM images were processed using Gwyddion software version 2.51 (Free Software Foundation, Boston, MA, USA) to obtain depth data. Water contact angles measurements were performed on the experimental surfaces using a model 100-00-230 NRL contact angle goniometer (Ramé-Hart Inc. Mountain Lakes, NJ, USA) in order to assess surface wettability. A small 5 µL MQ water droplet was applied on the surface. The average contact angle was measured based on seven measurements at 30 s time point.

### 2.3. Bacterial Preparation and Growth

*E. coli* strains BW25113 7636 (later referred as *E. coli-WT*), JW4277-1 11065 (ΔfimA, *E. coli*–Δ*fimA*), and JW4283-3 11068 (ΔfimH, *E. coli*–Δ*fimH*) (The Coli Genetic Stock Center, Yale University, New Haven, CT, USA) were grown in tryptic soy broth (TSB, Sigma-Aldrich, Oslo, Norway) medium overnight at 37 °C in centrifuge tubes and 5% CO_2_ atmosphere. *E. coli*–Δ*fimA* and *E. coli*–Δ*fimH* are mutant strains that lack the fimA and fimH genes, respectively. Such genes play a role in the production of fimbriae, and their parent is the *E. coli*–*WT* strain that has type 1 fimbriae [42]. The overnight culture was diluted 10 times the morning after and left to grow again in the same conditions until optical density reached OD_600_ = 1 (Thermo Scientific Spectronic 200E, Waltham, MA, USA). After that, samples were centrifuged at 5000 rpm at 21 °C to obtain a pellet. Medium residues were discarded and exchanged for PBS in order to obtain the same OD_600_ = 1. Afterwards, the pellet, in phosphate buffered saline (PBS, Lonza, Verviers, Belgium), was shaken, and the obtained solution was used later for injection. Average colony-forming units (CFUs) were measured by culturing bacteria overnight on TSB agar plates (overnight at 37 °C and 5% CO_2_ atmosphere). There was no observed significant difference in the number of colony forming units (CFU) at OD_600_ = 1 between the strains investigated. 

### 2.4. Bacterial Adhesion

**Figure 1:**
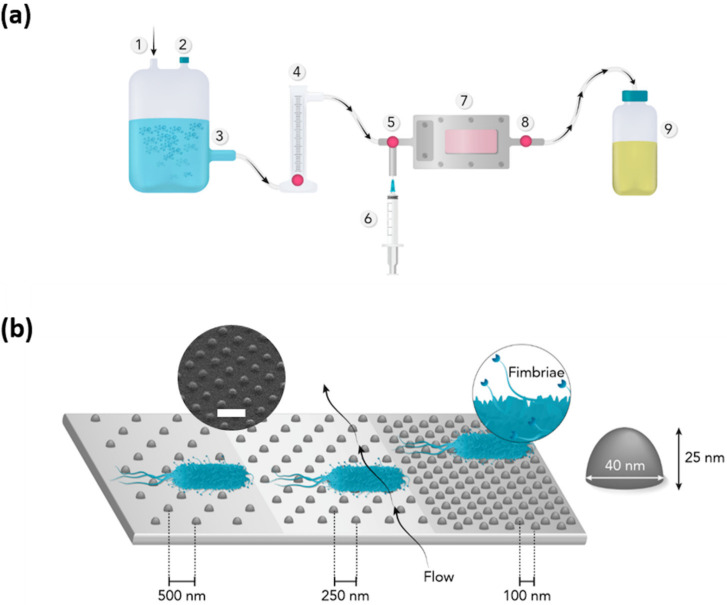
Flow chamber setup (**a**): 1—air supply, 2—air outlet, 3—distilled water tank, 4—flow meter, 5—right valve, 6—syringe input, 7—flow chamber with nanopatterned sample (*n* = 191), 8—left valve, 9—waste container; (**b**) graphical overview of tested surface (not in scale) with pillar height and width. Blue is bacteria. Arrows show direction of the flow. Interspace between pillars: 1–100 nm, 2–250 nm, 3–500 nm. Scanning electron microscope (SEM) image shows nanopattern (30° tilt) with scale bar = 200 nm.

After placing the sample in the flow chamber, the system was flushed with distilled water for about 1 min at a constant flow of 20 mL/min to remove any air bubbles trapped in the system. Then, 10 mL of bacteria in PBS was injected in the system manually using a syringe. The valves were then closed, and bacteria were let to adhere under static conditions for 5 min at room temperature. This procedure was followed by manually injecting 10 mL of 0.01% acridine orange (AO) to stain the cells for later viewing with fluorescence microscopy. After 3 min staining, valves were open again and sample was flushed for 5 min with distilled water at the same flow rate as before (20 mL/min). The setup is presented in Figure 1. Each strain was tested three times, and the total number of tested surface coverages was 191. 

### 2.5. Fluorescence Microscopy

For the counting of bacteria on the surfaces, the closed flow chamber, including glass cover, was transferred to a fluorescence light microscope (Olympus BX51, Olympus Optical, Tokyo, Japan). Pictures were taken at magnification of 90× by using a 10× magnification objective with U-MNB2 filter (excitation BP 470–490 nm and emission LP 520 nm), and one image was taken for each surface coverage for all replicas (*n* = 191).

### 2.6. Scanning Electron Microscopy

Samples were stored overnight in 2.5% glutaraldehyde buffered with 0.1 M Sørensen phosphate buffer, and afterwards were flushed with ethanol and PBS [43]. A field emission scanning electron microscope (SEM) was used to characterize the surfaces (Hitachi S-4800, Toyko, Japan). A thin layer of platinum (3 nm) was sputtered onto surfaces (Cressington 308R Coating System, Watford, UK), prior to SEM imaging. Pictures were taken at a magnification of 15k with the working distance (WD) set at 1.5 mm and acceleration voltage of 2.0 kV. The tilted image showing the nanopattern was taken at a magnification of 20k with the working distance set at 13.3 mm, acceleration voltage of 10 kV, and tilt set at 30°.

### 2.7. ImageJ Analysis

Image analysis was performed using ImageJ software version 1.53a (NIH, Bethesda, MD, USA). To calculate the coverage of the nanopattern, each picture was set to 8-bit, and the level of threshold was set to obtain a visible contrast between nanopattern and surface, which were later measured using the ”Analyze particles’’ plugin. To calculate the number of bacteria, we used an already established macro plugin [44], which allowed us to crop the original image in order to avoid artefacts from vignetting during the automated counting. The number of bacteria was then counted using the “find maxima” feature in the program, using a noise threshold of 12.

### 2.8. Statistical Analysis

Statistical analysis was performed using GraphPad Prism version 6.07 (GraphPad software, La Jolla, CA, USA). The effect of the different surface coverages for adhesion of the individual bacteria was analyzed using one-way ANOVA with repeated measurements followed by the Tukey’s multiple comparisons test. For analysis of the main effect of bacteria and surface coverage, a two-way ANOVA with repeated measurements (two factors in the experiment: surface coverage and bacteria strain) was performed followed by the Tukey’s multiple comparisons test for the simple effects within each column, and each bacterial strain compared to each other for each surface coverage was performed. For a subset of surfaces (*n* = 10) Students T-test was used to analyze differences in adhesion between surfaces without pillars compared to surfaces with the largest interpillar distance. Results were considered significant with a p-value ≤ 0.05. SPSS, version 25 (IBM Corp., New York, NY, USA), was used for calculating marginal means of bacteria number for all tested strains with significance at p-value ≤ 0.05. Total number of analyzed surface coverages was *n* = 191. Power analysis was conducted using G*Power version 3.1 (The G*Power Team, Düsseldorf, Germany). The effect size and total sample size were calculated with an alpha probability of 0.05 and power of 0.95 [45,46]. 

## 3. Results

### 3.1. Surface Characteristics

**Figure 2:**
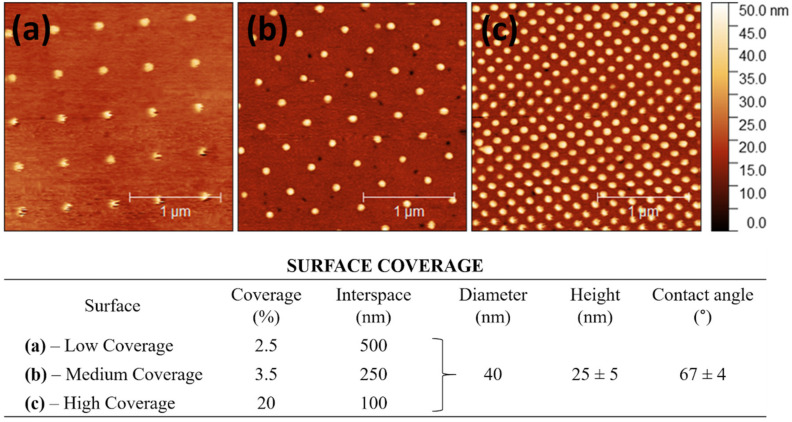
AFM images of surfaces with scale presented height (nm): (**a**) low coverage (LC), (**b**) medium coverage (MC), (**c**) high coverage (HC), and table below showing the characterization of the nanopillars with interspace, diameter, and height (*n* = 5).

Surfaces with injection molded 40 nm diameter features in polycarbonate were fabricated. Each pattern was divided into three sections with different surface coverages. The AFM measurements provided the interspace (100, 250, and 500 nm), pillar diameter of 40 nm, and pillar height of 25 ± 5 nm (Figure 2).

Wettability analysis along the surface showed that the characterized contact angle was smaller than 90° (after 30 s of measurement, an average contact angle was 67°), which means that surfaces used in our study were hydrophilic. Due to the small size of the nanopatterns (1 × 3 mm), we were limited to individual measurements of each nanopattern and present here only the overall surface contact angle.

### 3.2. Bacterial Adhesion

**Figure 3:**
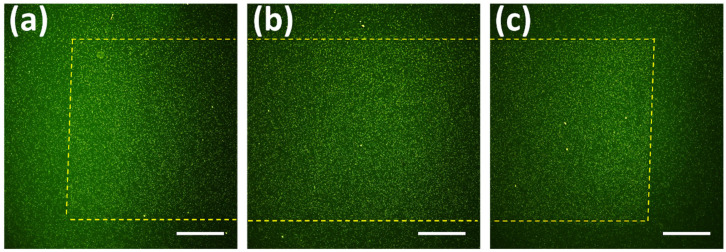
Fluorescent images of *E. coli-WT* after 5 min adhesion to different surface coverages at 10× magnification: (**a**) Low Coverage, (**b**) Medium Coverage, (**c**) High Coverage. The yellow dash marks the edges of the nanopattern. Green is bacteria. Scale bars = 250 µm.

**Figure 4:**
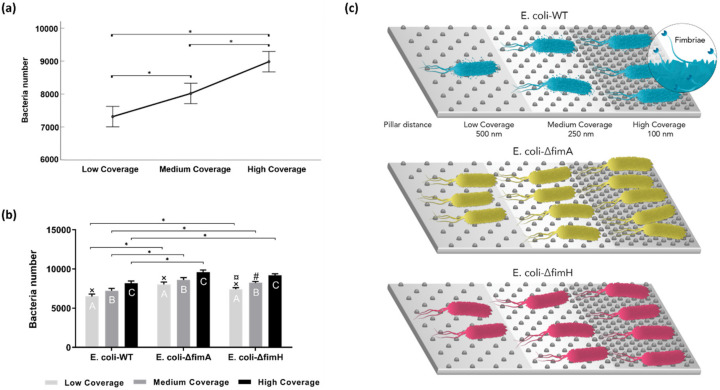
Bacteria adhesion to different surface coverages: (**a**) Bacteria number (calculated marginal means) for all tested strains. Significant at * *p*-value < 0.05. Median values with error bars represent the 95% confidence interval (*n* = 191, * *p* < 0.05); (**b**) average bacteria number to different surface coverages with the standard error of the mean. Comparison within tested strains (significant at *p*-value < 0.05, *n* = 191): × low coverage *vs.* medium coverage, # low coverage *vs.* high coverage, ¤ medium coverage *vs.* high coverage; (**c**) graphical presentation of tested strains attaching to different surface coverages.

Fluorescent microscopy images of adhered bacteria were used to quantify bacterial adhesion to the surfaces. An example of low coverage (Figure 3a), medium coverage (Figure 3b), and high coverage (Figure 3c) under fluorescence microscope is presented. Based on the power analysis, the calculated effect size was 0.29. Therefore, the calculated sample size was 186. In our study we used 191 surface coverages, which is higher than the calculated one. For each of the *E. coli*–*WT*, *E. coli*–Δ*fimA*, and *E. coli*–Δ*fimH*, increased adhesion with increasing surface coverage of nanopatterns was found (Figure 4a). The two-way ANOVA showed a significant main effect for both surface area coverage and the presence of type 1 fimbriae or not on adhesion. There was a significant increased adhesion for the mutants not expressing type 1 fimbriae compared to the wild type for each of the different surface coverages (low coverage, medium coverage, high coverage, Figure 4b). In order to compare an overall bacteria adhesion to different nanopattern surface coverages, the marginal means for all tested bacteria were calculated (Figure 4a). A graphical presentation of bacterial behavior on tested surface coverages is presented on Figure 4c.

**Figure 5:**
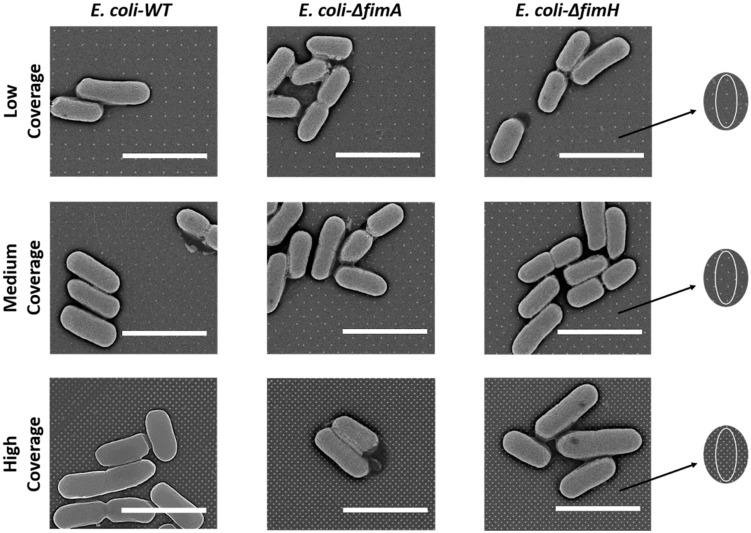
SEM images of tested bacteria. Fimbriae were not visible in all cases. Black arrows point at the theoretical position of bacteria (white oval) on the nanopattern. Scale bars = 2 µm.

Bacteria were observed to adhere to the nanopattern surfaces without losing their physiological morphology, keeping the characteristic rod shapes (Figure 5). No damaged bacteria cells were spotted during our study. The number of nanopillars in contact with one bacterium for the different surface coverages was calculated. Depending on the position of a single bacterium, the number of contact points for low coverage surfaces were 1–4 pillars, for medium coverage surfaces 8–10 pillars, and for high coverage surfaces approximately 45 pillars.

**Figure 6:**
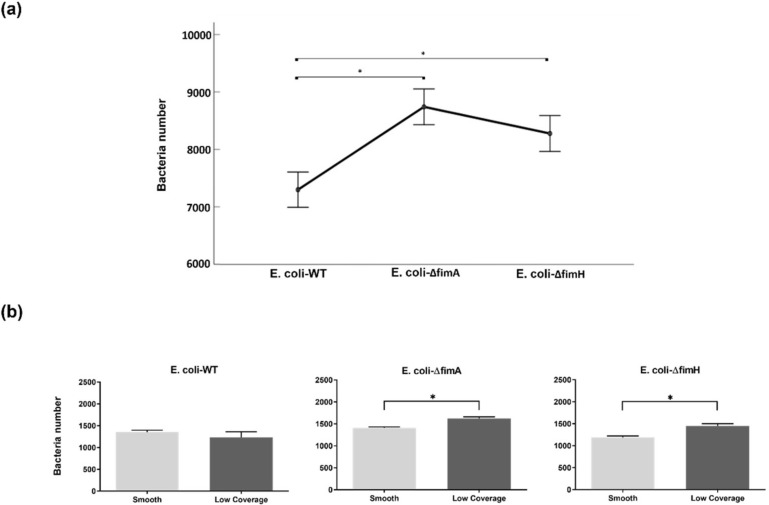
Effect of fimbriae on adhesion for all tested *E. coli* strains. (**a**) Bacteria number (calculated marginal means) significant at * *p*-value < 0.05. Median values with error bars represent the 95% confidence interval (*n* = 191, * *p* < 0.05). (**b**) Comparison within tested strains between smooth and low coverage. Average bacteria number to different surface coverages with the standard error of the mean. Significant at * *p*-value < 0.05, *n* = 10.

The effect of fimbriae on adhesion was observed by calculating and comparing marginal means (Figure 6a), where higher attachment was observed for *E. coli* without fimbriae. To analyze the effect of the presence of pillars, adhesion to areas without pillars, that is, smooth surfaces, was compared to areas with low coverage. Increased adhesion for the *fimA* and fim*H* mutants to the low coverage surfaces was observed compared to areas without pillars, whereas no difference was observed for the *E. coli*–*WT* (Figure 6b).

## 4. Discussion

Our work focused on investigating how nanopatterned surfaces affected the adhesion of *E. coli* and whether presence of fimbria would affect adhesion. Using a replication-based fabrication process, which combines an electron beam lithography and injection molding, we were able to create and examine a large number of surfaces with identical patterns and features. This process, as described in the previous work of Stormonth-Darling et al. [47], realizes the possibility of manufacturing almost any desired pattern and is a method considered to be a low cost and high speed alternative to other fabricating methods, such as poly dimethyl siloxane (PDMS) casting [48]. The electron beam lithography combined with injection molding method allows one to fabricate a large number of identical surfaces in a short time and at low cost. In comparison to other studies, often performed using few samples (typically with sample size of three) to investigate the different nano-patterned features [49,50,51], we used a larger sample size than the optimal sample size (*n* = 186, calculated by power analysis). When studying nano-patterned feature surface effects on bacteria, it is therefore important to always have the appropriate sample size. Unfortunately, when studying bacteria, optimal sample size is larger and it may be difficult to produce nano-patterned feature surfaces in large enough quantities.

The findings in the present study show that all strains investigated were affected by the presence of nanopillars on the surface. Although type 1 fimbriae is an important virulence factor for medical device infections, and expression is associated with increased biofilm formation and catheter-associated urinary tract infections [15,17,18,26,52,53], we observed reduced adhesion of the wild type *E. coli* compared to deletion mutants for the type 1 fimbriae monomers fimA or fimH for surfaces with nanopillars.

Our experimental findings showed an overall hydrophilic nature of the tested surfaces. Due to the size of the surfaces (1 × 3 mm), separate surface angle measurements for the different surfaces coverages were not performed. The hydrophilicity of surfaces has been described to affect bacterial adhesion. Otto et al. [54] used microscope slides in order to obtain hydrophilic (contact angle determined as <10°) and hydrophobic (contact angle determined as >85°) surfaces. They found that the expression of type 1 fimbriae by *E. coli* reduced initial adhesion compared to mutants not expressing fimbriae, in accordance with the present findings. In addition, the authors reported increased adhesion strength for *E. coli* expressing type 1 fimbriae to hydrophobic surfaces compared to hydrophilic surfaces, whereas no difference was observed for the *E. coli* not expressing type 1 fimbriae. 

Several studies have shown that adhesion of *E. coli* is affected by changes in surface topography. Widyaratih et al. [55] used electron beam induced deposition (EBID) in order to create silicon surfaces with four different nanopattern types. Their surfaces resembled osteogenic nanopatterns, and the adhesion of the *E. coli* K-12 strain was investigated. Topographies with tall nanopillars (over 100 nm) showed bactericidal effects and changes in the physiological morphology of cells. Among the changes were structure deformations and lack of ability to divide. For surfaces with nanopillars with lower heights (15–20 nm), comparable to the present study, a reduced adhesion was suggested compared to non-patterned surfaces, and no changes in morphology were observed (bacteria kept their rod shape). Another study by Bandara et al. [56] showed how natural nanotopography of dragonfly wing would affect adhesion of *E. coli* (NCTC 10418). Dragonfly wings are covered with nanopillars consisting of two populations with different heights, namely 189 ± 67 nm and 311 ± 52 nm, respectively. The presence of nanopillars led to damage of bacterial membrane as a result of strong adhesion between nanopillars and extracellular polymeric substances (EPS) from bacteria, as well as shear force which appeared during bacterial attempts to move on the nanopillars. In comparison, the nanopillars were approximately 4.7 and 7.5 times higher than the nanopillars in the present study. However, the height of the nanopillars is not the only factor affecting bacterial adhesion and survival on surfaces. Hasan et al. [57] fabricated surfaces inspired by the dragonfly wings based on deep reactive ion etching of a silicon wafer. The surfaces were highly hydrophobic with nanopillars of 4 nm height and 220 nm in diameter that exerted bactericidal activity against both *E. coli* and *Staphylococcus aureus*. 

In addition to the morphology of the nanopillars on the surfaces, the density of the nanopillars has been shown to affect both adhesive and bactericidal properties of surfaces. Dickson et al. [58] showed that by increasing the density of nanopillars on the surfaces of poly (methyl methacrylate) (PMMA) films, an increase in the bactericidal activity and decreased adhesion of *E. coli* was observed. The present study investigated surfaces with interspace distances varying between 100 and 500 nm. The higher the distance, the lower number of pillars that bacteria may adhere to. Depending on the cell size, the highest interspace distance would allow a single *E. coli* cell to establish contact with only one to four pillars. In contrast to the study by Dickson et al. [58], we observed increased initial adhesion to the surfaces with higher nanopillar density. This observation may be explained by, for example, a different contact time used to investigate adhesion and difference in chemistry of the surfaces

## 5. Conclusions

We have presented a method of producing a large number of identical nanostructured surfaces. *E. coli* wild type and deletion mutants of the type 1 fimbriae monomers FimA or FimH were tested for their ability to adhere to such surfaces with different interpillar distance of 100, 250, and 500 nm, respectively. An increase in interpillar distance (reduced surface coverage) was associated with reduced adhesion of *E. coli* wild type and the deletion mutants. In addition, the presence of a functional type 1 fimbria decreased adhesion to the nanopatterned surfaces in comparison to deletion mutants, leading us to the conclusion that there is a relationship between presence of a functional fimbriae and adhesion towards tested surfaces. The hypothesis that the presence of fimbriae had an effect on bacterial attachment to nanostructured surfaces was verified. These current results could provide insight into development of new nano-patterned structures with anti-adhesion bacterial properties.
